# Fine-Grained Intelligent Learning Diagnosis Model Based on the Exercise–Knowledge–Cognition Tensor for Educational Assessment

**DOI:** 10.3390/bs16050637

**Published:** 2026-04-24

**Authors:** Chunyan Zeng, Yulin Hou, Zhifeng Wang

**Affiliations:** 1 Hubei Key Laboratory for High-Efficiency Utilization of Solar Energy and Operation Control of Energy Storage System, Hubei University of Technology, Wuhan 430068, China; 2 Faculty of Artificial Intelligence in Education, Central China Normal University, Wuhan 430079, China

**Keywords:** learning diagnosis, taxonomy of educational objectives, educational sustainability, cognitive levels, machine learning

## Abstract

Accurate and interpretable learning diagnosis is increasingly required in AI-enabled educational assessment. Existing cognitive diagnostic models typically represent item attributes with a binary Q-matrix and infer mastered or not mastered knowledge states. Although polytomous extensions allow graded mastery, item attributes rarely encode theory-aligned cognitive-process demands, which limits pedagogical interpretation of diagnosed profiles. This study aims to operationalize revised Bloom’s taxonomy at the exercise–knowledge level by constructing an Exercise–Knowledge–Cognition tensor and to develop RLDM-EKC as a DINA-type cognitive diagnosis model that infers ordered knowledge–cognition profiles. The model defines EKC-based ideal responses, estimates slip and guess parameters with an Expectation–Maximization procedure, and derives learner profiles using Maximum A Posteriori inference with uncertainty summaries. We validate the approach on synthetic data and on TIMSS 2007 Grade 4 mathematics data, comparing against classical CDMs including DINA, PA-DINA, and pG-DINA. In simulation, RLDM-EKC attains a PMR of 81.7% and an AAMR of 91.6%, and in empirical data, it yields theory-aligned multi-level cognitive profiles with transparent uncertainty reporting. These properties support actionable, human-in-the-loop feedback for teachers and learners under realistic deployment constraints.

## 1. Introduction

With the rapid development of society and the continuous advancement of information technology, the landscape of education is undergoing profound transformations (Omodan, 2024). In AI-enabled learning platforms, smart education increasingly leverages artificial intelligence and big data to support teaching and assessment processes, which raises the demand for diagnostic models that provide precise and interpretable feedback beyond total scores (L. Li et al., 2026; Z. Zhang & Yang, 2020). At the same time, learners are exhibiting a growing demand for precise learning support and personalized feedback on their development. Traditional test methods that rely solely on total scores are no longer sufficient to meet the deeper needs of educational assessment. Consequently, there is an urgent need for diagnostic approaches capable of analyzing learners’ internal cognitive processes and providing a fine-grained depiction of their knowledge mastery, thereby enabling precision teaching tailored to individual differences. Against this backdrop, learning diagnosis—an interdisciplinary field integrating psychometrics, educational psychology, and intelligent algorithms—has gradually become a pivotal area of research in smart education (Wang et al., 2023; Wu et al., 2024).

Learning diagnosis typically focuses on the analysis of internal variables such as learners’ knowledge mastery, use of learning strategies, and cognitive processes. When centered on cognitive learning and knowledge skills, learning diagnosis is also regarded as an application of cognitive diagnosis within educational assessment (Zhan, 2021). Modeling approaches represented by cognitive diagnostic models (CDMs) (Wang et al., 2024) can integrate learners’ response data and exercise attributes to characterize cognitive features at the knowledge concept level. These models rely on the structural relationships between exercises and knowledge concepts, commonly represented by a Q-matrix, where each row indicates the knowledge concepts assessed by an exercise, and each element takes a binary value of 0 or 1. Based on the Q-matrix and response data, researchers have developed a variety of typical CDMs, such as DINA (De La Torre, 2009), G-DINA (Ma & De La Torre, 2020), and LCDM (Henson et al., 2009). These models not only determine whether a learner has mastered a specific knowledge concept, but also reveal patterns of knowledge mastery and learning deviations. However, current research primarily concentrates on modeling and analyzing dichotomously scored exercises (i.e., 0/1 scoring). Although certain extended models incorporate the concept of multi-level knowledge mastery to analyze the degree to which a learner understands a given knowledge concecpt, several challenges remain: (1) there is a lack of in-depth modeling of the relationship between exercise content and cognitive levels, making it difficult to align cognitive diagnostic results with established educational and cognitive theories; (2) the models often lack corresponding information from the domains of education and cognitive science, resulting in limited interpretability of the inferred cognitive states and inadequate support for the formulation of precise learning strategies; (3) the outputs of these models are still difficult to translate into actionable learning recommendations. These limitations hinder the effectiveness of CDMs in providing evidence-based attribution of learners’ cognitive issues. As a result, learners may have insufficient understanding of their own knowledge and cognitive profiles, and consequently lack targeted guidance for planning and adjusting their learning strategies. This can lead to reduced learning efficiency and increased learning costs. Therefore, there is an urgent need to incorporate more systematic cognitive frameworks to enhance the interpretability and practical applicability of the models. Accordingly, this study aims to encode revised Bloom’s cognitive-process levels into exercise attributes at the exercise–knowledge granularity and to infer learners’ ordered knowledge–cognition profiles in a DINA-type framework with explicit uncertainty reporting.

To address the aforementioned challenges, this study builds on Bloom’s Taxonomy of Educational Objectives (Bloom, 1964) and adopts its revised cognitive-process taxonomy (Revised Bloom’s taxonomy) to operationalize hierarchical cognitive levels (Remembering, Understanding, Applying, Analyzing, Evaluating, and Creating) (Anderson & Krathwohl, 2001), thereby constructing a novel three-dimensional structure—the Exercise–Knowledge–Cognition (EKC) tensor. Building upon the traditional exercise–knowledge associations, the EKC tensor further integrates the cognitive levels assessed by each exercise. This integration allows the incorporation of domain-specific educational knowledge into algorithmic modeling at the data level, thereby enhancing both the theoretical grounding and cognitive interpretability of the model. On this basis, we propose a Refined Learning Diagnosis Model based on the EKC tensor (RLDM-EKC). The primary novelty lies in the EKC representation and its integration into the ideal-response definition to enable theory-aligned multi-level cognitive profiling, while the underlying response mechanism remains DINA-type with slip and guess parameters. This model integrates the ideal response mechanism and response function formulation of DINA-type models and employs both the Expectation–Maximization (EM) algorithm (Byrne & Eggermont, 2011) and Maximum A Posteriori (MAP) estimation (Tronarp et al., 2021). By jointly leveraging learners’ response data and the EKC structure, the RLDM-EKC model enables fine-grained and interpretable modeling of learners’ knowledge mastery from a cognitive perspective. Experiments on both synthetic and real-world datasets show that RLDM-EKC achieves competitive diagnostic performance and model-data fit relative to established CDMs, while producing theory-aligned multi-level cognitive profiles with uncertainty information. This provides a practical basis for personalized learning support and for downstream instructional decision-making.

The remainder of this paper is organized as follows: Section 2 provides a brief review of related work; Section 3 introduces relevant task definitions and outlines the research objectives of this study; Section 4 details the implementation of the proposed RLDM-EKC model; Section 5 presents experimental analyses based on synthetic and real datasets; finally, Section 6 concludes the paper.

## 2. Related Work

In this section, we review the related work on refined learning diagnostic methods that encompasses the following three main areas: the Q-matrix theory, classification of educational objectives, and cognitive diagnosis.

### 2.1. Classification of Educational Objectives

#### 2.1.1. Traditional Classifications and Their Limitations

Educational objectives have traditionally been categorized into three domains: facts, skills, and attitudes. This classification, valued for its simplicity and ease of use, has seen widespread adoption among educators. However, it also has notable limitations. The categories are conceptually broad, making them difficult to operationalize in teaching and assessment, which often leads to biased evaluations. Additionally, the boundaries between these domains are vague, lacking clear logical hierarchies, which hampers the precise representation of instructional goals (Zainuddin et al., 2023). While this traditional framework no longer dominates educational research, it has inspired the development of more systematic and scientifically grounded classification theories.

#### 2.1.2. Representative Frameworks for Educational Objectives

Since the 20th century, psychologists and educators have proposed various influential classification systems that have significantly advanced educational theory and improved instructional practice. British educational psychologist Rowntree (1974) categorized educational objectives into survival skills, method objectives, and content objectives, emphasizing their alignment with real-world needs and individual development in social contexts. Subsequently, American psychologist Bloom et al. (1956) introduced a more structured taxonomy, classifying objectives into cognitive, affective, and psychomotor domains. This framework laid a theoretical foundation for instructional design and learning assessment. Further contributions came from Gagne et al. (2005), who proposed five categories of learning outcomes—verbal information, intellectual skills, cognitive strategies, attitudes, and motor skills—based on the nature of learning results. He also emphasized the temporal distinction between educational objectives (pre-instruction) and learning outcomes (post-instruction), positioning learning outcomes as concrete manifestations of educational goals. These classification systems have evolved to reflect increasing complexity, systematization, and adaptability in educational objective research.

#### 2.1.3. Bloom’s Taxonomy of Cognitive Objectives

This study adopts the Revised Bloom’s Taxonomy (RBT) (Anderson & Krathwohl, 2001) as the theoretical framework for describing the cognitive demand of assessment tasks. RBT was proposed by Anderson and Krathwohl and further summarized by Krathwohl, and it reconceptualizes educational objectives through a two-dimensional framework: a knowledge dimension (factual, conceptual, procedural, and metacognitive knowledge) and a cognitive-process dimension (Remember, Understand, Apply, Analyze, Evaluate, and Create). In contrast to the original taxonomy, the revised framework uses action-oriented categories to better align learning objectives, assessment design, and instructional practices.

Recent studies have increasingly operationalized the revised Bloom’s taxonomy in data-rich learning settings to support scalable labeling, analytics, and feedback loops. For example, Almatrafi and Johri (2025) investigated GPT-4 prompt strategies for classifying instructor-authored learning outcomes into cognitive-process levels in the revised taxonomy, demonstrating the feasibility of AI-assisted large-scale tagging with agreement analysis against expert annotations. Along similar lines, Holl et al. (2024) released a higher-education question dataset with meta-features explicitly annotated using the revised Bloom’s taxonomy, enabling downstream modeling and analysis that distinguishes higher-order from lower-order cognitive demands. In learning analytics and hybrid-intelligence settings, Y. Lee et al. (2025) leveraged revised Bloom-aligned cognitive-process categories to interpret behavioral cues in digital reading and to infer learners’ internal cognitive processes. Meanwhile, Larsen et al. (2022) empirically examined core assumptions behind the revised taxonomy (e.g., knowledge-type vs. cognitive-process dimensions and the role of action verbs), highlighting the need to report labeling reliability and validity evidence when Bloom-level tags are used as analytical variables. Finally, Y. Li et al. (2022) demonstrated that Bloom-level labels for learning objectives can be learned from text at scale using modern NLP models, supporting practical adoption when manual expert coding is costly. However, these studies also imply an important measurement constraint: once Bloom-level labels are used as analytical variables, their reliability and construct validity become part of the psychometric argument rather than a purely descriptive coding step. In other words, the challenge is not only to assign cognitive-process labels at scale, but to embed them in a diagnostic framework in a way that preserves interpretable ordered meaning and admits validity checks. This consideration is central to the present study, which treats expert-tagged Bloom levels as model-defining item attributes and therefore reports both inter-rater reliability and external construct validity evidence.

### 2.2. Cognitive Diagnosis

Cognitive diagnosis is a core component of the next generation of test theory (Mislevy, 2021). It integrates advancements from psychometrics and cognitive psychology based on traditional test theory (Xin et al., 2022). The goal is to deeply assess an individual’s cognitive processes, processing skills, and knowledge structure. The central idea is to establish a relationship between test items and knowledge points. By analyzing a learner’s responses to test items through cognitive diagnostic models (CDMs), we can examine the learner’s latent scores, learning state, cognitive situation, and the quality of the items (Williamson, 2023). Accurate construction of a cognitive diagnostic model is crucial for analyzing the underlying cognition and the quality of test items behind a learner’s score. It serves as a bridge linking the learner’s external test responses with their internal learning cognition.

#### 2.2.1. Cognitive Diagnosis Based on Item Response Theory

Item Response Theory (IRT) uses mathematical modeling to explain and predict the relationship between a test taker’s ability and their responses. It models test items in greater detail, investigating how item characteristics (such as difficulty and discrimination) affect the learner’s responses. Currently, cognitive diagnosis based on IRT is evolving into a model that combines the continuous latent variables of traditional IRT with discrete mastery patterns of attributes. This approach extends the application of IRT, balancing information measurement with cognitive diagnostic accuracy, and facilitates more flexible and practically valuable cognitive diagnostic tasks.

To address the binary scoring issue in traditional IRT and cognitive diagnostic models, S. Lee and Gu (2024) proposed the general-response CDM. By expanding the scope of IRT, this model handles various response types, including continuous, count, and response time data, which are common in modern educational assessments. However, this model depends on the precise specification of the Q-matrix, and the diversity of response types makes the interpretation of diagnostic results relatively complex. To address the model’s dependence on the Q-matrix and to tackle challenges in multi-dimensional and multi-attribute contexts, Liu et al. (2025) proposed a model that combines general-response CDM with higher-order latent variables. This model not only processes multiple response types but also allows for simultaneous estimation of test parameters and learner latent attributes, even in the absence of a known Q-matrix. This expands the application of IRT in cognitive diagnosis. However, this method is more complex and requires large sample sizes, presenting challenges in computational cost and empirical validation in practical applications. To overcome the limitation of traditional CDMs, which separate ability from cognitive attributes, Song et al. (2024) introduced a method that combines the ability from IRT as a covariate with CDM. This allows the model to handle both continuous ability estimates and discrete cognitive attributes simultaneously, providing more detailed diagnostic results. However, the model’s accuracy may be affected when error and guessing parameters of the items are large.

Overall, research on cognitive diagnosis based on IRT is advancing toward greater flexibility and precision. However, it faces challenges such as computational complexity, model interpretability, and practical feasibility. A key future direction in this field will be how to balance the model’s flexibility with its interpretability, address computational issues when handling large-scale data, and expand the application of these models in broader educational and psychological assessments.

#### 2.2.2. Cognitive Diagnosis Based on Cognitive Attributes

Cognitive diagnosis based on cognitive attributes models treats cognitive attributes as the basic unit, connecting test items with latent attributes through a pre-set or estimated Q-matrix. This allows for the inference of learners’ attribute mastery patterns (i.e., “mastered/not mastered”) based on their responses. With the increasing complexity of educational test structures, ability requirements, and cognitive measurement goals, research on cognitive diagnosis based on cognitive attributes focuses on addressing the subjectivity and potential mis-specifications of Q-matrix settings, the uncertainty of attribute hierarchies, and the dependence of traditional CDMs on simple binary responses or basic knowledge points.

To reduce reliance on expert-subjective Q-matrix and hierarchy settings, Y. Chen and Wang (2023) proposed a Bayesian method that allows the model to recover attribute hierarchies and CDM parameters from response data, rather than relying entirely on expert priors. This enhances the flexibility of CDMs in testing attribute structures in unknown or new domains. Subsequently, to address the risk of mis-specification that may arise from Q-matrices set by experts, Liu and Wu (2023) proposed a Q-matrix correction method based on a complete-information matrix. This method uses statistical tests to systematically identify and correct potential errors in the Q-matrix, improving the accuracy of attribute recovery and the reliability of the Q-matrix. To further handle cases where both the Q-matrix and attribute hierarchies might be mis-specified, Wang and Sun (2025) introduced a joint verification mechanism that combines Bayesian methods with conditional independence tests. This approach corrects both the Q-matrix and hierarchy assumptions, strengthening the robustness of CDM at the structural level. Finally, to address the potential failure of traditional hierarchy validation methods (such as likelihood-ratio tests) under non-standard conditions, X. Zhang et al. (2025) proposed a statistical testing method for attribute hierarchies using Wald statistics (Wald-XPD/Wald-Obs), providing new research ideas for validating hierarchy structures and model selection.

At the same time, the applicability of cognitive diagnostic models based on cognitive attributes is also expanding. To cope with real-world testing scenarios involving multiple attributes, multiple levels, large test banks, and large sample sizes, Han et al. (2025) proposed a two-stage polytomous attribute estimation method that can handle large-scale tests with numerous items and multi-level attributes, overcoming the limitations of traditional binary attribute models in large-scale tests. Additionally, to improve the reliability of diagnostic estimates and avoid the overly optimistic estimates caused by traditional point-estimation methods, Kreitchmann et al. (2023) introduced multiple imputation (MI) into CDM. By considering the parameter uncertainty of the model, this approach makes the classification results more robust.

In summary, research on cognitive diagnosis based on cognitive attributes is gradually progressing towards directions such as Q-matrix correction, multi-level attribute large-scale testing, and robust reliability estimation. These advancements enhance the credibility and applicability of CDM.

#### 2.2.3. Cognitive Diagnosis Based on Computerized Adaptive Testing

Computerized Adaptive Testing (CAT) is a testing mechanism that dynamically and individually selects test items to achieve maximum measurement precision with the minimum test length. Combining CAT with cognitive diagnostic models (CDMs) helps accurately diagnose learners’ mastery of multiple knowledge or skill attributes (attribute mastery) while reducing testing time and the number of items. This approach balances both efficiency and the depth of diagnostic information (Nájera et al., 2025).

Current research on adaptive learning for cognitive diagnosis tends to integrate methodical exploration with practical application and systematic implementation in real educational settings. For example, Y. Li et al. (2023) developed a CD-CAT system for reading comprehension assessments in elementary schools and conducted empirical testing with over 20,000 students, demonstrating significant advantages in diagnostic accuracy and efficiency with the CD-CAT framework. However, this method requires a high-quality item pool and calibration data; if the item pool or calibration data is insufficient, its advantages may not be as evident. To address this, Cao et al. (2024) optimized the item selection mechanism at the algorithmic level, formalizing the item-selection problem in CD-CAT as a graph path search problem. This improvement enhanced item pool utilization balance and the stability of classification. Additionally, to increase test flexibility, J. Zhang et al. (2024) proposed a “chunking program” method, which implemented item re-review and answer modification functions within each chunk, thus enhancing the flexibility of nonparametric CD-CAT.

In the early stages of adaptive testing, the system may struggle to estimate the learner’s ability and cognitive state accurately due to a lack of sufficient response data. To address this “cold start” issue, Liu et al. (2025) proposed a FACD framework, which combines collaborative and personalized modules to accelerate the diagnostic accuracy and convergence speed of CD-CAT during the early stages, significantly improving diagnostic accuracy at the beginning of the CAT process. As research progresses, the application of CD-CAT is expanding to more educational contexts. For example, Morphew et al. (2024) applied CD-CAT to STEM teaching through the LASSO platform, showcasing its potential for classroom assessment. Y. Zhang et al. (2025) constructed a comprehensive CD-CAT system that integrates item pool construction, Q-matrix generation, model calibration, and recommendation system integration, demonstrating a feasible path for embedding CD-CAT into teaching platforms.

In summary, current research reflects the trend of CD-CAT evolving from conceptual exploration to practical implementation, making it a promising tool in the fields of smart education, personalized learning, and teaching feedback.

Taken together, the reviewed CDM literature shows that recent advances have improved multi-level attribute estimation, Q-matrix verification, and adaptive administration, but they still treat item attributes primarily as statistical requirement structures rather than as theory-encoded cognitive-process demands. In parallel, revised Bloom’s taxonomy has been increasingly operationalized for labeling, analytics, and learning-objective classification, yet this line of work has rarely been structurally embedded into a cognitive diagnosis framework in which item attributes, ideal responses, and learner profiles are jointly defined by ordered cognitive requirements. This gap motivates RLDM-EKC. Instead of introducing a new response family, we incorporate an EKC-based item representation into a DINA-type diagnosis framework to infer theory-aligned, multi-level knowledge–cognition profiles together with uncertainty summaries.

## 3. Preliminaries

In this section, we will provide an overview of the entire fine-grained learning diagnostic approach and provide relevant problem definitions. The main symbols of the paper and their corresponding descriptions are presented in Table 1.

### 3.1. Exercise–Knowledge–Cognition Tensor

**Figure 1:**
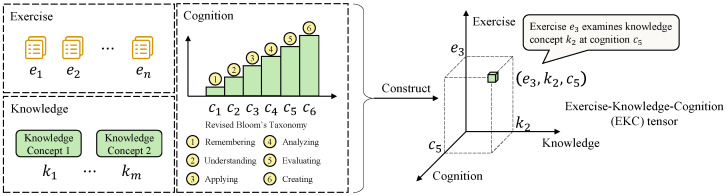
Schematic of the EKC tensor.

Traditional learning diagnosis models primarily rely on an exercise–knowledge Q-matrix to specify whether an exercise requires a knowledge concept. This representation is adequate for binary attribute linkage, but it does not encode the minimum cognitive-process level at which the concept must be mobilized. To address this structural limitation, we adopt the Revised Bloom’s Taxonomy (RBT) (Radmehr & Drake, 2019) in the cognitive domain and construct an Exercise–Knowledge–Cognition (EKC) tensor, whose structure is illustrated in Figure 1. Formally, let $A={\left\{A_{ekc}\right\}}_{E\times K\times C}$, where $A_{ekc}=1$ indicates that exercise *e* requires knowledge concept *k* at cognitive level *c*, and $A_{ekc}=0$ otherwise. Under the finalized annotation protocol, each exercise–knowledge pair is assigned at most one positive cognitive level; therefore, the tensor can be equivalently summarized by an ordered requirement map $a_{ek}\in \left\{0,1,\cdots ,6\right\}$, where $a_{ek}=0$ means that concept *k* is not required by exercise *e* and $a_{ek}=c$ means that concept *k* is required at Bloom level *c*. This representation provides a modeling advantage beyond extending the Q-matrix: it preserves concept involvement and simultaneously encodes the minimum ordered cognitive requirement needed for a theoretically correct response. As a result, the EKC structure directly constrains ideal-response formation, parameter estimation, and ordered profile inference, making cognitive-process interpretation part of the model specification rather than a purely post hoc description. In what follows, *E*, *K*, and *C* denote the exercise, knowledge, and ordered cognitive-level spaces, respectively.

### 3.2. Problem Formulation

Consider *U* learners responding to *E* exercises. Let $R=R_{ue}$ denote the binary response matrix, where $R_{ue}=1$ if learner *u* answers exercise *e* correctly and $R_{ue}=0$ otherwise. Each exercise is associated with an EKC representation that specifies, for every knowledge concept k∈K, the ordered cognitive level required by that exercise. Under this setting, the target of the model is not only to estimate exercise-level slip and guess parameters, but also to infer each learner’s ordered knowledge–cognition profile $\beta_{u}=\left(\beta_{u1},\cdots ,\beta_{uK}\right)$, where $\beta_{uK}\in \left\{0,1,\cdots ,6\right\}$.

**Problem Definition**: Given the response matrix *R* and the EKC representation *A*, estimate exercise parameters and recover each learner’s multi-level knowledge–cognition profile across knowledge concepts.

## 4. Fine-Grained Learning Diagnosis Model Based on the Exercise–Knowledge–Cognition Tensor

In this section, the proposed RLDM-EKC model is presented, which introduces educational domain knowledge from the data level by constructing the EKC tensor to achieve a fine-grained portrayal and interpretable modeling of learners’ knowledge mastery state at the cognitive level. Methodologically, RLDM-EKC should be understood as a DINA-type cognitive diagnosis model: it retains the slip/guess response mechanism, but replaces a binary item-requirement representation with EKC-based ordered cognitive requirements that define ideal responses over multi-level knowledge–cognition profiles.

### 4.1. Overall Framework of the Model

**Figure 2:**
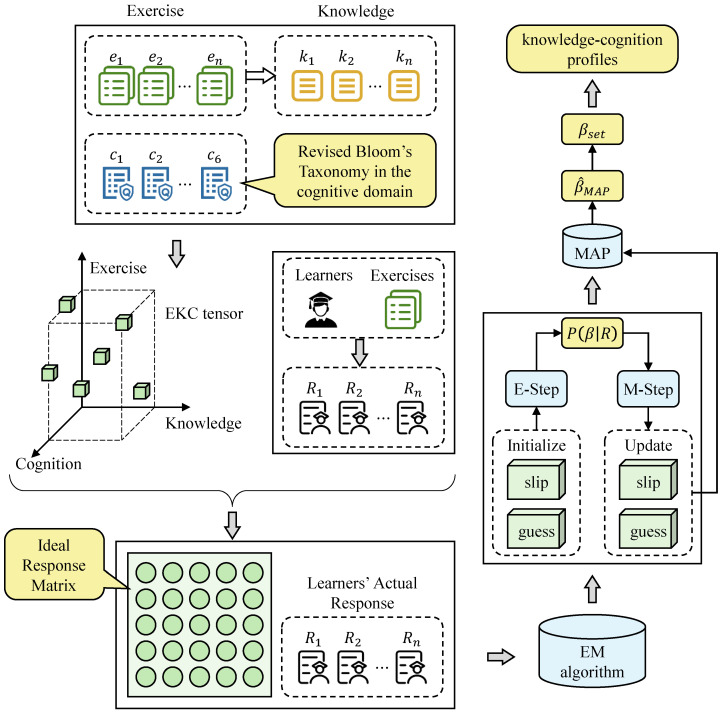
Framework of the fine-grained learning diagnosis model.

The fine-grained learning diagnosis model based on the EKC tensor integrates revised Bloom’s taxonomy in the cognitive domain with principles from learning diagnosis. Leveraging the highly interpretable EKC tensor, the model first combines it with learners’ actual response data to calculate their ideal response scores. Then, the Expectation–Maximization (EM) algorithm is employed to estimate exercise parameters. Subsequently, based on the estimated exercise parameters, learner response functions are constructed to compute the probability of each learner responding correctly under all possible cognitive states. These probabilities are then used to build a candidate set of cognitive levels for each learner using the Maximum A Posteriori (MAP) estimation. Finally, a global expectation is calculated across all cognitive state vectors in the candidate set, and a comprehensive decision is made to determine each learner’s final knowledge cognitive level. The overall framework of the fine-grained learning diagnosis model based on the EKC tensor is illustrated in Figure 2.

### 4.2. Construction of the Fine-Grained Learning Diagnosis Model

The fine-grained learning diagnosis model utilizes the constructed EKC tensor and learners’ actual response data to estimate exercise parameters and diagnose learners’ cognitive proficiency levels. These cognitive levels align with the hierarchical structure defined by revised Bloom’s taxonomy in the cognitive domain. The specific algorithmic procedure of the model is described as follows.

First, learners’ response data are preprocessed, and invalid exercises (answered correctly or incorrectly by all learners) are removed. Then, we construct the EKC tensor through a reproducible expert-tagging protocol: three subject-matter experts independently assign an ordinal cognitive level to each exercise–knowledge unit following a shared guideline; a short briefing and calibration step is conducted to align interpretations of the level definitions before formal coding. The experts were selected to represent senior domain expertise in the assessment subject and demonstrated familiarity with curriculum-based item review and revised Bloom’s taxonomy. Before formal coding, we conducted a structured training and calibration procedure that included a detailed walkthrough of the annotation guideline, discussion of representative examples, and a consensus check to standardize level interpretations across experts. After independent annotation, ratings are consolidated to obtain a single EKC tensor, where agreements are retained and remaining disagreements are resolved through an explicit adjudication procedure to ensure labeling quality. We further quantify the consistency of the pre-adjudication annotations via inter-rater reliability (IRR), reporting Krippendorff’s α for ordinal data and pairwise quadratic weighted kappa (QWK) with nonparametric bootstrap 95% confidence intervals (1000 resamples). The ordinal Krippendorff’s α is 0.715 (95% CI: 0.552–0.828), and the pairwise QWK values are 0.765, 0.847, and 0.739 (mean 0.784, 95% CI: 0.666–0.864). In addition, the exact agreement among all three experts is 0.632, and agreement within ±1 level reaches 0.985. Finally, the consolidated annotations are used to form the complete EKC tensor. As an external criterion for construct validity, we subsequently examine whether these Bloom-based cognitive-level requirements align with the TIMSS cognitive domains (Knowing/Applying/Reasoning) at the item level. Because the EKC tensor is expert-tagged, residual labeling noise can propagate to both exercise-parameter estimation and learner-profile inference. If the required cognitive level is systematically overestimated, more correct ideal responses become misclassified as incorrect, which tends to increase the estimated slip rate. If the required level is underestimated, more incorrect ideal responses become misclassified as correct, which can inflate the estimated guessing rate and shift posterior mass toward higher mastery levels. We mitigate these effects by reporting pre-adjudication reliability, applying adjudication to obtain a single tensor, and explicitly reporting posterior uncertainty for inferred levels, while treating remaining tagging noise as a limitation.

Then, for the possible $7^{K}$ cognitive states of each learner, an indicator function I(·) is used to compute the learner’s ideal response matrix π. The ideal response of learner *u* to exercise *e*, denoted as $\pi_{ue}$, is defined as

(1) $$\pi_{ue}=\prod_{k=1}^{K}I\left(A_{EKC}\leq \beta_{uk}\right)$$

(2) $$I\left(A_{EKC}\leq \beta_{uk}\right)\left\{\begin{array}{cc} 1 & A_{EKC}\leq \beta_{uk}\\ 0 & A_{EKC}>\beta_{uk} \end{array}\right.$$

Equations (1) and (2) indicate that, when considering only the learner’s cognitive ability, if the cognitive level $\beta_{uk}$ on any knowledge concept *k* of learner *u* is lower than the cognitive level required by exercise *e* on that knowledge concept, then the learner *u* is theoretically expected to answer exercise *e* incorrectly, i.e., $\pi_{ue}=0$. Conversely, suppose the cognitive level of learner *u* on all relevant knowledge concepts meets or exceeds the levels required by the exercise *e*. In that case, the learner *u* is theoretically expected to answer exercise *e* correctly, i.e., $\pi_{ue}=1$.

Furthermore, based on the actual response $R_{u}$ of learner *u* and the ideal response $\pi_{u}$, the slip parameter $s_{e}$ and guess parameter $g_{e}$ for exercise *e* are defined as follows: if the ideal response of learner *u* is correct but the actual response is incorrect, a slip is considered to have occurred; if the ideal response is incorrect but the actual response is correct, a guess is assumed. The mathematical expressions of these two parameters are given as

(3) $$\begin{array}{c} s_{e}=P\left(R_{ue}=0|\pi_{ue}=1\right)\\ g_{e}=P\left(R_{ue}=1|\pi_{ue}=0\right) \end{array}$$

Then, the response probability that learner *u* with cognitive mode $\beta_{n}$ will answer correctly on exercise *e* is denoted as

(4) $$P_{e}\left(\beta_{n}\right)=P\left(R_{ue}=1|\beta_{n}\right)=g_{t}^{\left(1-\pi_{ue}\right)}{\left(1-s_{e}\right)}^{\pi_{ue}}$$

Assuming that learner *u* has a knowledge–cognition profile denoted by $\beta_{u}$, and that the learner’s responses to different exercises are conditionally independent, the conditional distribution response vector $R_{u}$ of learner *u* is given by

(5) $$L\left(R_{u}|\beta_{u}\right)=\prod_{e=1}^{E}P_{e}{\left(\beta_{u}\right)}^{R_{ue}}{\left[1-P_{e}\left(\beta_{u}\right)\right]}^{1-R_{ue}}$$

Accordingly, the conditional distribution of the response data *R* for all learners is

(6) $$L\left(R|\beta \right)=\prod_{u=1}^{U}L\left(R_{u}|\beta_{u}\right)=\prod_{u=1}^{U}\prod_{e=1}^{E}P_{e}{\left(\beta_{u}\right)}^{R_{ue}}\left[1-P_{e}{\left(\beta_{u}\right)}^{1-R_{ue}}\right]$$

If the learner’s knowledge–cognition profile $\beta_{u}$ is known, the exercise parameters can be directly estimated. However, in practice, the cognitive profile $\beta_{u}$ is typically unknown and must be inferred. Therefore, it is necessary to consider the marginal likelihood function of the learner’s response behavior. The specific computation is given in Equation (7):

(7) $$L\left(R\right)=\prod_{u=1}^{U}L\left(R_{u}\right)=\prod_{u=1}^{U}\sum_{n=1}^{N}L\left(R_{u}|\beta_{n}\right)P\left(\beta_{n}\right)$$

Here, $L\left(R_{u}\right)$ denotes the marginal likelihood of the response vector $R_{u}$ of learner *u*, and $P\left(\beta_{n}\right)$ represents the prior probability of the cognitive profile $\beta_{n}$. The index $1\leq n\leq 7^{K}$ corresponds to all possible knowledge–cognition profiles when there are *K* knowledge concepts. Since Equation (7) involves the latent variable $\beta_{n}$, it is not feasible to perform direct maximum likelihood estimation. Because the number of candidate profiles grows as $7^{K}$, exact enumeration is feasible for the low-dimensional TIMSS subsets studied here but may become computationally restrictive as *K* increases. We therefore use exact posterior evaluation as a transparent baseline in the present setting, while recognizing that larger-scale applications may require approximate inference or additional structural constraints. In addition, the likelihood factorization in Equation (5) relies on the standard local-independence assumption of CDMs: conditional on a learner’s latent knowledge–cognition profile, responses are treated as independent across exercises. This assumption supports tractable estimation and interpretable parameterization, but it may be violated in testlets, shared-stimulus designs, or other locally dependent assessment settings and should therefore be regarded as a modeling simplification. Therefore, the RLDM-EKC model employs the Expectation–Maximization (EM) algorithm to perform parameter estimation:

E-step: Using the initialized or previously estimated values of the slip parameter $s_{e}$ and the guess parameter $g_{e}$, calculate $P\left(R|\beta \right)={\left[P\left(R_{u}|\beta_{n}\right)\right]}_{U\times N}$ and use the marginal likelihood $P\left(R|\beta \right)$ to calculate the values in the matrix $P\left(\beta |R\right)={\left[P\left(\beta_{n}|R_{u}\right)\right]}_{N\times U}$:

(8) $$P\left(\beta_{n}|R_{u}\right)=\frac{P\left(R_{u}|\beta_{n}\right)P\left(\beta_{n}\right)}{P\left(R_{u}\right)}=\frac{P\left(R_{u}|\beta_{n}\right)P\left(\beta_{n}\right)}{\sum_{n=1}^{7^{K}}P\left(R_{u}|\beta_{n}\right)}$$

Here, $P\left(\beta_{n}|R_{u}\right)$ denotes the posterior probability that learner *u* belongs to the cognitive profile $\beta_{n}$.

M-step: Based on the E-step, the expected number of learners possessing the cognitive profile $\beta_{n}$ is denoted as $Z_{n}=\sum_{n=1}^{7^{K}}P\left(\beta_{n}|R_{u}\right)$. The expected number of learners with cognitive profile $\beta_{n}$ who correctly answered exercise *e* is denoted as $F_{en}=\sum_{n=1}^{7^{K}}P\left(\beta_{n}|R_{u}\right)R_{ue}$. Then, by setting $\frac{\partial \log L\left(R\right)}{\partial g_{e}}=0$ and $\frac{\partial \log L\left(R\right)}{\partial s_{e}}=0$, the slip and guess parameters are updated as ${\hat{g}}_{e}=\frac{F_{en}^{0}}{Z_{en}^{0}}$ and ${\hat{s}}_{e}=\frac{Z_{en}^{1}-F_{en}^{1}}{Z_{en}^{1}}$, respectively. Here, $F_{en}^{0}$ represents the expected number of learners with cognitive profile $\beta_{n}$ who answered exercise *e* correctly despite having at least one dimension of knowledge below the required level for that exercise, that is, guessers. $F_{en}^{1}$ represents the expected number of learners with a cognitive profile $\beta_{n}$ who fully meet the level of knowledge required by exercise *e* and answered correctly, i.e., ideal responders. Similarly, $Z_{en}^{0}$ denotes the expected number of learners with profile $\beta_{n}$ whose knowledge mastery is incomplete for exercise *e*, and $Z_{en}^{1}$ denotes those who fully meet the cognitive requirements for exercise *e*. For each exercise, the total expected number of learners with cognitive profile $\beta_{n}$ is given by $Z_{n}=Z_{en}^{0}+Z_{en}^{1}$. Thus, using the posterior estimates computed in the E-step, one can derive $F_{en}^{0}$, $Z_{en}^{0}$, $F_{en}^{1}$, and $Z_{en}^{1}$, and subsequently update the estimates of ${\hat{s}}_{e}$ and ${\hat{g}}_{e}$. The detailed procedure for estimating exercise parameters using the EM algorithm is shown in Algorithm 1.
**Algorithm 1:** Estimation of exercise parameters
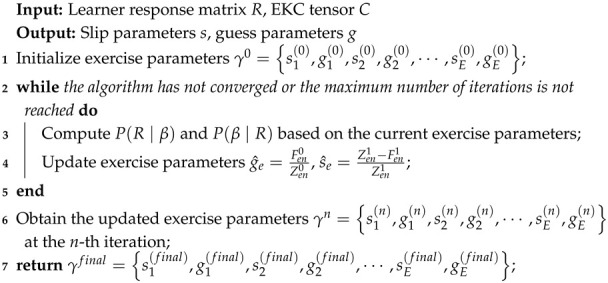


Based on the exercise parameters $\gamma^{final}$ estimated using the EM algorithm, the most probable knowledge–cognition profile $\beta_{n}$ for learner *u*, given their response vector $R_{u}$, is obtained from the posterior distribution:

(9) $${\hat{\beta }}_{MAP}=\underset{1\leq n\leq 7^{K}}{\arg\max}\;P\left(\beta_{n}|R_{u}\right)$$

We adopt ${\hat{\beta }}_{MAP}$ as a point estimate, while explicitly retaining the full posterior $P\left(\beta_{n}|R_{u}\right)$ to quantify statistical uncertainty in the inferred profiles. The tie-handling rule leverages the ordinal nature of Bloom-aligned levels to avoid arbitrary selection among equally probable profiles, and the rounding step provides an interpretable discrete level while preserving posterior uncertainty for transparency. When multiple profiles share the same maximum posterior probability, they are collected into a candidate set $\beta_{set}$, and a global expectation is computed across these candidates; the resulting expected level is then rounded by interval decision rules and mapped to the ordered levels in revised Bloom’s cognitive taxonomy (0 for not mastered; 1–6 for the six hierarchical levels). Importantly, the posterior information also enables uncertainty reporting at the attribute level: for each learner *u* and knowledge concept *k*, we compute the marginal posterior over the seven ordered levels and summarize it with a 95% credible interval. In addition, we conduct an adjacent-level separability diagnostic using a representative neighboring pair (Level 2 vs. Level 3; Understanding vs. Applying), and report evidence-based indeterminacy rates and boundary-crossing rates. Algorithm 2 outlines the specific steps for inferring learners’ knowledge–cognition levels.
**Algorithm 2:** Inference of learners’ knowledge–cognition profiles
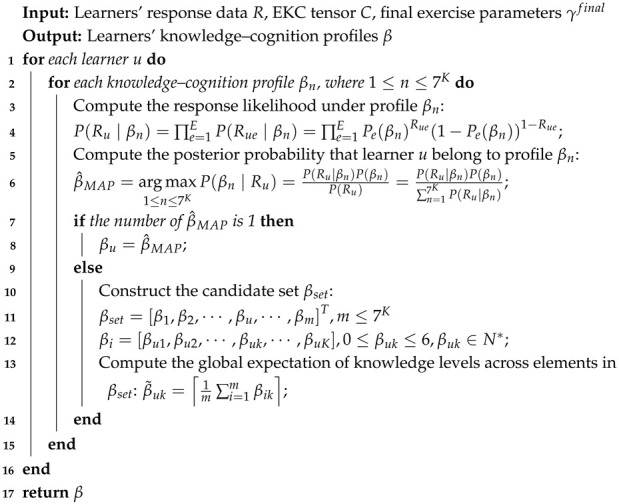


**Figure 3:**
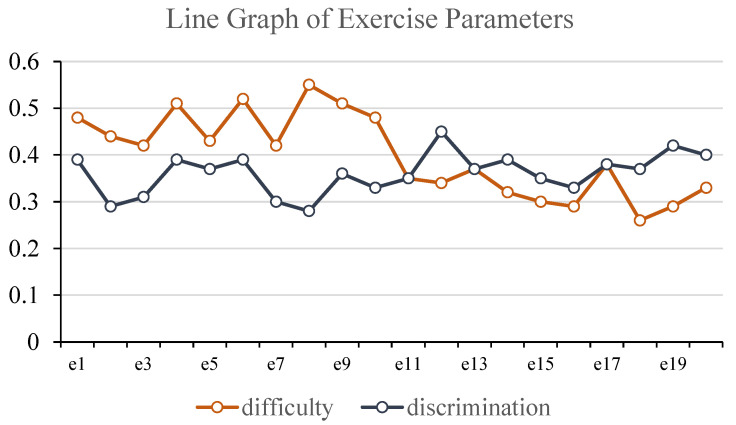
Line graph of exercise parameters.

**Figure 4:**
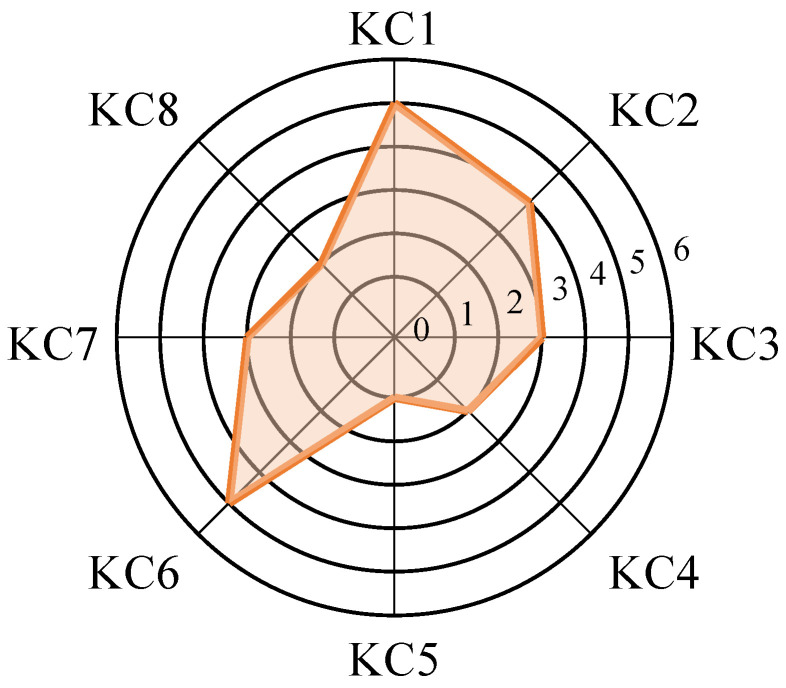
Radar chart of learners’ cognitive levels.

After estimating the exercise parameters and inferring learners’ knowledge–cognition levels using the fine-grained learning diagnosis model based on the EKC tensor, the output results are visualized for intuitive interpretation. Visualization is implemented using the Python 3.12 libraries Pygal 3.0 and Matplotlib 3.9. Specifically, line charts are employed to depict the slip and guess parameters for each exercise, which help evaluate exercise difficulty and the overall test structure. This provides a basis for targeted adjustments to exercises in accordance with the intended assessment objectives. In addition, radar charts are used to illustrate each learner’s cognitive level across different knowledge dimensions. This visualization offers a clear and intuitive representation of individual learners’ cognitive states, facilitating the identification of learning weaknesses and enabling focused training interventions. Examples of the above two types of visualizations are shown in Figure 3 and Figure 4, respectively.

## 5. Experiments and Analyses

In this section, we evaluate the effectiveness of the proposed fine-grained learning diagnosis method, RLDM-EKC, in uncovering learners’ cognitive patterns over knowledge concepts. We begin by outlining the key research questions addressed by the experiments, followed by a detailed description of the datasets and their sources. Subsequently, we compare the proposed model with several classical models in the field of cognitive diagnosis. We include DINA as a binary-mastering baseline and pG-DINA and PA-DINA as representative models that support multi-level attributes. On synthetic data, we report both exercise-parameter recovery using MAE and MSE and profile-recovery accuracy using PMR and AAMR to align with standard CDM simulation practice. On TIMSS data, we report AIC and BIC for model-data fit, while interpreting fit indices together with the intended goal of producing theory-aligned cognitive profiles with uncertainty information. Finally, we conduct experiments on both synthetic and real-world datasets, and analyze the results to validate the model’s performance. The experiments are designed to address the following research questions:

*RQ1*: How well does the proposed RLDM-EKC model perform in uncovering learners’ cognitive patterns over knowledge concepts on synthetic data?

*RQ2*: Compared with classical cognitive diagnostic models, how does the RLDM-EKC model perform in uncovering learners’ cognitive patterns over knowledge concepts on real-world data?

### 5.1. Dataset

The experiments in this study are conducted using data from the Trends in International Mathematics and Science Study (TIMSS), a large-scale international assessment organized by the International Association for the Evaluation of Educational Achievement (IEA) to monitor global education quality. TIMSS evaluates the mathematics and science achievement of fourth- and eighth-grade students across approximately 20 countries and has been conducted every four years since 1995. The 2007 TIMSS dataset is widely used in educational research and provides comprehensive information, including detailed exercise content, student response scores, evaluation outcomes, and SPSS 29.0 analysis syntax. Sample exercise information is illustrated in Table 2, where “M” indicates mathematics exercises and “S” indicates science exercises. If the key column states “See scoring guide,” the exercises is open-ended and requires reference to a scoring rubric. For multiple-choice exercises, “A” indicates the correct option, while other options such as “B,” “C,” and “D” represent distractors. The dataset contains actual learner response records, exercise metadata, and fully specified exercise attribute patterns. In subsequent experiments, we exploit the independence assumption between knowledge concepts—namely, a learner’s cognitive state on one concept is unaffected by their mastery of others. This allows for both partial and complete data configurations to be used in targeted validation experiments. In the empirical evaluation on real-world data, we use the fourth-grade mathematics subset from the 2007 TIMSS dataset. We follow the preprocessing described in Section 4.2 by removing invalid exercises that are answered correctly or incorrectly by all learners. For open-ended items, responses are binarized according to the TIMSS scoring guide to match the binary-response setting. For the multi-step constructed-response exercises noted in Section 5.3, partially correct responses are treated as incorrect to ensure a consistent response format across exercises.

### 5.2. Empirical Evaluation Based on Simulated Data (RQ1)

**Figure 5:**
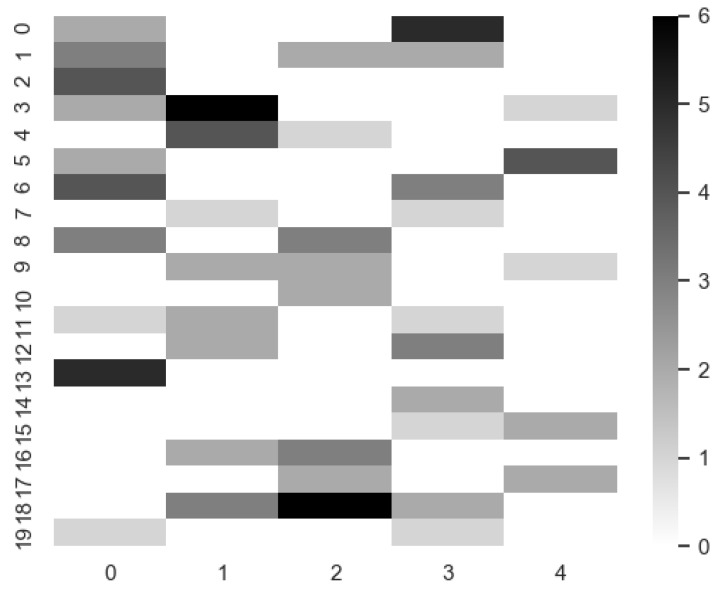
Heat map of the EKC tensor used for the simulation experiments.

This study employs a Monte Carlo simulation (Paganetti, 2025) approach to evaluate the effectiveness and accuracy of the fine-grained learning diagnosis model based on the EKC tensor. First, the EKC tensor required for the experiment is generated according to a Gaussian distribution, with the overall mean cognitive level for each knowledge concept set to 2 and the variance set to 3. To provide a more intuitive understanding of the EKC tensor, a heat map visualization is used, as illustrated in Figure 5. The EKC tensor represents the cognitive requirements of 20 exercises across 5 knowledge concepts. Based on this tensor, knowledge–cognition profiles for 20,000 simulated learners are generated, following a Gaussian distribution. In total, there are $7^{5}$ possible cognitive profiles. Slip and guess parameters for each exercise are initialized using a four-parameter beta distribution, with values drawn from the interval [0.1, 0.4]. The probability of learner *u* answering exercise *e* correctly is calculated using Equation (4). Learners’ response scores are then simulated using Bernoulli sampling based on this probability. As a result, ground truth data are obtained for the EKC tensor, exercise parameters, learner knowledge–cognition profiles, and simulated response scores. The entire data generation and simulation process is repeated 100 times to conduct a comprehensive simulation-based evaluation. We choose Gaussian distributions to generate heterogeneous yet centered cognitive levels, which produces a broad range of mastery patterns without forcing extreme corner cases. The beta distribution range for slip and guessing parameters is set to avoid degenerate near-deterministic responses and to reflect a moderate level of response noise under which diagnostic differences among models are observable.

The estimation accuracy of exercise parameters is evaluated using Mean Absolute Error (MAE) and Mean Square Error (MSE) (Hodson, 2022). MAE provides an interpretable average absolute deviation, while MSE penalizes larger deviations more strongly. Lower values of both MAE and MSE indicate that the estimated parameters are closer to the ground truth. The formulas for these two metrics are given in Equations (10) and (11).

(10) $$MAE\left(y_{i},{\hat{y}}_{i}\right)=\frac{1}{m}\sum_{i=1}^{m}\left|y_{i}-{\hat{y}}_{i}\right|$$

(11) $$MSE\left(y_{i},{\hat{y}}_{i}\right)=\frac{1}{m}\sum_{i=1}^{m}{\left(y_{i}-{\hat{y}}_{i}\right)}^{2}$$

The accuracy of the inferred learners’ knowledge–cognition profiles is evaluated using Pattern Match Ratio (PMR) (CAI et al., 2013) and Average Attribute Match Ratio (AAMR) (Z. Luo, 2019). PMR measures the proportion of learners whose entire cognitive profile is correctly identified, while AAMR evaluates the average proportion of correctly inferred cognitive attributes across all learners. Higher values of PMR and AAMR indicate greater accuracy in diagnosing learners’ knowledge–cognition levels. These metrics are widely used for CDM parameter-recovery and attribute-classification evaluation in simulation studies, which enables a direct and interpretable assessment of estimation and diagnosis quality under controlled ground truth (F. Luo et al., 2023; Tu et al., 2012). The computation of these two metrics is defined in Equations (12) and (13).

(12) $$PMR=\frac{\sum_{u=1}^{U}N_{u\_correct}}{U}$$

(13) $$AAMR=\frac{\sum_{u=1}^{U}\sum_{k=1}^{K}N_{uk\_correct}}{U\times K}$$

In this evaluation, if the cognitive profile of learner *u* is correctly identified, then $N_{u\_correct}=1$; similarly, if the cognitive level of learner *u* on the knowledge concept *k* is correctly inferred, then $N_{uk\_correct}=1$.

Based on the EKC tensor and the experimental settings described above, the corresponding dataset is generated, and the simulation experiment is repeated 100 times. The final results are averaged over all iterations. Table 3 presents the experimental results on simulated data, including a detailed comparison between the estimated and true values of exercise parameters across all exercises, as well as the overall Mean Absolute Error (MAE) and Mean Square Error (MSE) of the exercise parameters. Additionally, the table reports the Pattern Match Ratio (PMR) and Average Attribute Match Ratio (AAMR) for all learners. The results indicate that the average MAE of the estimated slip and guess parameters *s* and *g* over 100 runs is below 0.04, and the MSE is below 0.02—both within acceptable error margins. Furthermore, the model achieves a PMR of 81.7% and an AAMR of 91.6%, demonstrating that the fine-grained learning diagnosis model based on the EKC tensor not only provides highly accurate exercise parameter estimates (i.e., guess and slip rates) but also achieves high accuracy in diagnosing learners’ cognitive profiles. In conclusion, the simulation results validate the effectiveness of the proposed EKC-based fine-grained learning diagnosis model in both exercise parameter estimation and learner knowledge–cognition diagnosis, thereby supporting its applicability to real-world educational assessment scenarios.

### 5.3. Empirical Evaluation Based on Real-World Learning Data (RQ2)

To further validate the effectiveness of RLDM-EKC in practical assessment scenarios, we conduct experiments on a real-world dataset. The empirical data are drawn from the 2007 Trends in International Mathematics and Science Study (TIMSS) fourth-grade mathematics assessment, as used in the study by Yamaguchi and Okada (2018). The publicly available Q-matrix is decomposed into three binary (0/1) submatrices corresponding to the domains of “Number,” “Geometric Shapes & Measures,” and “Data Display” (Y.-S. Lee et al., 2011). Based on the test content, domain experts in education annotate the EKC tensor according to revised Bloom’s taxonomy. The annotated EKC tensor is then aligned and calibrated against the Q-matrix to ensure accuracy. For clarity, a quantized example of the EKC tensor is shown in Table 4. Using response data from learners in the United States (excluding Minnesota and Massachusetts) as well as from selected schools in Minnesota and Massachusetts (Yamaguchi & Okada, 2018), we evaluate the performance of RLDM-EKC on real assessment data. Table 5 presents summary statistics for the dataset. Note that for subjective exercises M041258 (steps A and B) and M031242 (steps A, B, and C), partially correct responses are considered incorrect. To assess the model’s fit to real-world data, we adopt two standard model selection criteria: the Akaike Information Criterion (AIC) (Akaike, 2025) and the Bayesian Information Criterion (BIC) (Neath & Cavanaugh, 2012). Lower values of AIC and BIC indicate better model-data fit.

The model fitting results for different models across datasets are shown in Table 6. In addition to the DINA model, we include two comparative methods: the pG-DINA model proposed by J. Chen and de la Torre (2013) and the PA-DINA model proposed by Cai and Tu (2015). Experimental results indicate that the proposed fine-grained learning diagnosis model achieves better data-model fit than both the DINA and PA-DINA models. Compared with pG-DINA, RLDM-EKC shows comparable fit across the three subsets: it is slightly worse in AIC/BIC on Datasets 1 and 3, while achieving a slightly better AIC (with a marginally worse BIC) on Dataset 2. Importantly, RLDM-EKC is designed to yield more consistent and interpretable multi-level cognitive profiles, which supports instructional diagnosis beyond overall fit indices. Table 7 presents the knowledge–cognition profiles inferred by each model for the 172nd learner in Dataset 1. The PA-DINA and pG-DINA models can identify the attained mastery level, yet their interpretability is limited without an explicit cognitive framework. In contrast, the DINA model only provides a binary mastered/not-mastered judgment and cannot infer fine-grained levels. The proposed RLDM-EKC model offers interpretable diagnostic results at a finer granularity by incorporating revised Bloom’s taxonomy to represent ordered cognitive levels, yielding profiles that are comparable to those of PA-DINA and pG-DINA. Beyond point estimates, we further quantify statistical uncertainty in the inferred cognitive levels and examine whether neighboring levels can be empirically distinguished. Specifically, we test a representative adjacent pair, Level 2 vs. Level 3 (Understanding vs. Applying), which is well-supported by boundary items in TIMSS Grade 4 mathematics. As shown in Table 8, we define an identifiability gate using the availability of Level 2 and Level 3 boundary items for each knowledge concept (gate = Strong if $n_{q2}\geq 2$ and $n_{q3}\geq 2$, otherwise Weak), and we summarize adjacent-level separation among cases with MAP∈{2,3} by $n_{2/3}$, Indet. (the proportion whose 95% credible interval spans both Levels 2 and 3), Med.|log-odds| with $\log-\mathrm{odds}=\log(p_{u,k}^{\left(2\right)}/P_{u,k}^{\left(3\right)})$, and P(|Δ|<0.10) with $\Delta =p_{u,k}^{\left(2\right)}-p_{u,k}^{\left(3\right)}$, where $p_{u,k}^{\left(2\right)}$ and $p_{u,k}^{\left(3\right)}$ denote the marginal posterior probabilities of Levels 2 and 3. In Dataset 1, KC2 is identifiable (gate = Strong, $n_{q2}=2$, $n_{q3}=2$) and shows low indeterminacy for 2/3 among learners with MAP∈{2,3} ($n_{2/3}=388$, indeterminate rate = 0.090, median |log-odds| = 2.251, P|Δ|≤0.10=0.003), while KC4 also shows strong separability (gate = Weak, $n_{q2}=1$, $n_{q3}=2$, $n_{2/3}=888$, indeterminate rate = 0.034, median |log-odds| = 4.682, P|Δ|≤0.10=0.016). In contrast, KC3 exhibits substantial ambiguity (gate = Weak, $n_{q2}=6$, $n_{q3}=1$, $n_{2/3}=228$, indeterminate rate = 1.000, median |log-odds| = 0.007, P|Δ|≤0.10=0.965), consistent with insufficient boundary evidence. In Dataset 2, KC2 is strongly separable (gate = Strong, $n_{q2}=3$, $n_{q3}=3$, $n_{2/3}=113$, indeterminate rate = 0.000, median |log-odds| = 8.632, P|Δ|≤0.10=0.000), whereas in Dataset 3, the only testable concept (KC1, gate = Weak, $n_{q2}=3$, $n_{q3}=1$) is not separable for 2/3 ($n_{2/3}=183$, indeterminate rate = 1.000, median |log-odds| = 0.005, P|Δ|≤0.10=1.000). Collectively, these results indicate that RLDM-EKC can produce fine-grained, theory-aligned cognitive profiles while also providing transparent uncertainty reporting: when boundary evidence is sufficient, adjacent levels (2/3) are distinguishable with strong posterior evidence; when boundary evidence is weak, the model appropriately reflects high uncertainty rather than over-claiming separability.

### 5.4. Interpretable Analysis

In the modeling process of cognitive and learning diagnosis, the interpretability of a model is a critical criterion for evaluating its practical value and instructional applicability. The proposed RLDM-EKC model not only incorporates the theoretical foundation of revised Bloom’s taxonomy in the cognitive domain, but also explicitly models the cognitive requirement relationships between items and knowledge concepts through the construction of the Exercise–Knowledge–Cognition (EKC) tensor. This significantly enhances the educational interpretability and practical usability of the model’s diagnostic outcomes.

Firstly, from the perspective of exercises, the EKC tensor incorporates cognitive level labels, enabling each exercise to be associated not only with its corresponding knowledge concepts but also with the depth of cognitive processing required for each knowledge concept within the exercise. This modeling approach transcends the binary limitation of traditional Q-matrices—i.e., whether a knowledge concept is assessed—by refining the representation of cognitive demands embedded in exercise design. It facilitates teachers’ analysis of the functional positioning of items at different knowledge levels and supports subsequent targeted adjustments to test structures and instructional content. Secondly, from the learner perspective, the RLDM-EKC model jointly models response data and the EKC structure to generate predictions of each learner’s cognitive level across different knowledge concepts. The results are expressed using a seven-level scale ranging from “Unmastered (0)” to the six categories of the Revised Bloom’s Taxonomy. This output adheres to an established educational theory framework and allows teachers to hierarchically interpret learners’ cognitive processes, forming a closed-loop cognitive feedback system from “Remembering” to “Understanding”, “Applying”, “Analyzing”, “Evaluating”, and “Creating”. The interpretability of the model is thereby significantly enhanced. Furthermore, as illustrated in Figure 4, the learner cognitive radar chart visualizes the distribution of cognitive levels across multiple knowledge concepts. This chart intuitively reflects learners’ cognitive structural characteristics and helps instructors quickly identify cognitive weaknesses and areas of strength, thus providing a data-driven basis for personalized instruction. When combined with the model’s cognitive level outputs, it also supports the generation of automated personalized learning reports, enhancing learners’ metacognitive awareness and self-regulation abilities. Finally, the guessing and slipping parameters in the RLDM-EKC model also offer strong interpretability. As shown in the example in Figure 3, the model estimates the distribution of guessing and slipping rates across items, which can reveal underlying issues such as insufficient exercise discrimination or measurement error in test design. These insights enable instructors to identify items prone to excessive guessing or disproportionate difficulty, thus providing valuable references for exercise revision and evaluation standard setting, and further promoting the scientific and standardized development of assessment tools.

In summary, the RLDM-EKC model demonstrates strong interpretability in multiple aspects, including the modeling of exercise-level cognitive hierarchies, the characterization of learners’ cognitive levels, and the visualization of diagnostic results. These features not only enhance the model’s instructional applicability and educational credibility, but also provide robust support for the development of high-quality intelligent learning diagnosis.

### 5.5. Construct Validity Analysis

To further establish the construct validity of the Bloom-based cognitive level annotations encoded in the EKC tensor, we utilize the well-validated TIMSS cognitive domain framework (Knowing, Applying, Reasoning) as an external criterion. Specifically, we aim to verify whether the fine-grained cognitive demand requirements reflected by our expert-tagged EKC tensor are consistent with the broader cognitive domain classifications defined by TIMSS, which would provide empirical support for the educational interpretability of our cognitive diagnosis framework.

TIMSS organizes items from a cognitive-demand perspective into three broad cognitive domains (Knowing, Applying, and Reasoning). To examine whether the Bloom-based cognitive-level requirements encoded in our expert-tagged EKC tensor are construct-aligned with this domain perspective, we first define a transparent crosswalk between the two frameworks: Bloom levels 1 and 2 are mapped to TIMSS Knowing, levels 3 and 4 to TIMSS Applying, and levels 5 and 6 to TIMSS Reasoning. We then summarize item-level cognitive demand using $c_{max}$, defined as the maximum Bloom level among the non-zero EKC entries required by an item across all involved knowledge concepts. We then compare $c_{max}$ across domains using the Kruskal–Wallis test (with $\epsilon^{2}$ as a nonparametric effect-size index) and quantify the monotonic trend using Spearman correlation between the ordered domain index (Knowing = 1, Applying = 2, Reasoning = 3) and $c_{max}$. Table 9 presents the domain-wise distributions of $c_{max}$ (reported as median and interquartile range, IQR) and mean item proportion-correct across datasets and TIMSS cognitive domains, while Table 10 summarizes the corresponding construct validity test statistics.

As shown in Table 9, $c_{max}$ increases systematically from Knowing to Applying and further to Reasoning in Dataset 1: the median $c_{max}$ shifts from 2.0 (Knowing) to 3.0 (Applying) and 5.0 (Reasoning), with a significant between-domain difference (Kruskal–Wallis H=15.017, $p=5.48\times 10^{-4}$, $\epsilon^{2}=0.814$) and a strong monotonic trend (ρ=0.913, $p=4.79\times 10^{-8}$) as reported in Table 10. A consistent increase is also observed in Dataset 2, where items are distributed across Knowing and Applying (no Reasoning items in this subset): H=5.600, p=0.0180, $\epsilon^{2}=0.767$, and ρ=0.894, p=0.0027). For Dataset 3, the monotonic ordering remains evident (ρ≈1.000, p≪0.001), while the omnibus test is not significant at α=0.05 (H=5.000, p=0.0821), which is expected given the small number of items in this subset.

Notably, the cognitive-demand signal captured by $c_{max}$ is not reducible to item difficulty in these data: the association between $c_{max}$ and the item proportion-correct is weak and non-significant across subsets (all p>0.65), and proportion-correct does not show a reliable between-domain difference (all p>0.16), suggesting that the Bloom-based levels primarily reflect intended cognitive demand rather than mere item easiness or hardness.

### 5.6. Human-in-the-Loop Deployment and Educational Implications

To align the proposed RLDM-EKC with a human-in-the-loop application perspective, we emphasize how the diagnostic outputs can be transferred to real educational scenarios in schools and universities through a practical workflow that explicitly involves teachers and learners. In deployment, the item bank is first aligned with knowledge concepts and cognitive-process categories through EKC tagging, after which learner response logs are used to generate actionable, uncertainty-aware diagnostic reports that summarize learners’ knowledge–cognition profiles and flag items with abnormal guessing/slipping tendencies. For teachers, these reports support instructional decision-making by identifying concepts where learners remain at lower cognitive processes and by guiding targeted remediation, while for learners, the same information can be communicated as descriptive, non-technical feedback that facilitates self-regulated learning and practice planning. Effective use requires lightweight training opportunities for educators to interpret both “level” and “uncertainty” without overgeneralization and to adapt teaching practices from score-focused feedback toward process-oriented support, and it also raises ethical considerations that should be addressed through data minimization, purpose limitation, transparent communication, and human review in high-uncertainty or high-stakes cases to avoid labeling effects and protect students’ privacy. While the proposed visualizations and the construct validity analysis provide structural evidence that Bloom-aligned cognitive levels are interpretable as intended, the present study does not include a formal user study with teachers or assessment experts to quantify how diagnostic reports are understood, trusted, and acted upon in authentic instructional routines. We therefore treat downstream interpretability and instructional impact as an open evaluation dimension and encourage future work to assess agreement, usability, and decision quality under realistic classroom constraints.

## 6. Conclusions and Future Work

Cognitive diagnosis models play a critical role in the new generation of educational measurement theory, serving as a vital bridge between learners’ internal cognitive processes and their external exercise response behaviors for educational sustainability. In traditional assessment settings, CDMs can accurately, efficiently, and quickly extract valuable information about exercises and uncover learners’ underlying knowledge states. Although numerous CDMs have been developed by scholars, few models are specifically designed to assess the cognitive level of learners. To address the limitations of traditional CDMs—such as coarse diagnostic granularity, shallow inference, and lack of interpretability—this paper proposes a Refined Learning Diagnosis Model based on the Exercise–Knowledge–Cognition tensor. The model introduces the EKC tensor, which integrates the conventional exercise–knowledge Q-matrix with revised Bloom’s taxonomy in the cognitive domain, providing interpretable insights from an educational psychology perspective. It further leverages learners’ actual response data to estimate exercise parameters and infer learners’ cognitive levels. Empirical evaluations on both simulated and real-world datasets demonstrate that RLDM-EKC enables effective parameter estimation and reliable cognitive diagnosis. The model supports multi-dimensional analysis and can deeply explore the diverse cognitive profiles of learners. This refined diagnostic granularity facilitates more accurate and informative learning assessments. From a pedagogical perspective, the resulting knowledge–cognition profiles can help teachers identify concepts on which learners remain at relatively low cognitive processes and make more targeted instructional adjustments or remediation plans. The same outputs can also be communicated to learners as process-oriented, non-technical feedback that supports study planning, self-regulated learning, and a clearer understanding of how knowledge mastery relates to cognitive demand. Beyond improving interpretability at the reporting level, the theoretical contribution of RLDM-EKC is to incorporate hierarchical cognitive structures into CDM at the model-specification level. By encoding revised Bloom-aligned cognitive requirements in the EKC tensor and integrating them into the ideal-response definition, the model extends traditional binary attribute diagnosis to ordered knowledge–cognition profiling. In this sense, RLDM-EKC does not merely attach pedagogical labels to CDM outputs; rather, it makes hierarchical cognitive demand part of the latent representation that links item requirements, response formation, and learner profile inference.

Moreover, in the current version of the proposed fine-grained learning diagnosis model, the learner response matrix is binary-valued, where each element indicates either a correct (1) or incorrect (0) response. As a result, the model performs well in cognitive diagnosis for objective exercises but lacks the capacity to deeply analyze subjective exercises with polytomous (multi-level) scoring. Furthermore, the final diagnosis of learners’ cognitive profiles is based on an expectation-based inference method, which implicitly assumes that the distribution of cognitive levels across knowledge concepts tends to follow a Gaussian-like pattern. This assumption may lead to deviations in diagnosing certain learners’ true knowledge–cognition states. Therefore, a pressing direction for future work is to explore how polytomous scoring data from subjective exercises can be effectively integrated with the EKC tensor, enabling accurate diagnosis of learners’ cognitive levels in open-ended or complex tasks. This extension would further enhance the integration of cognitive diagnosis models with educational practice, promote personalized learning, and ultimately improve the effectiveness of learning diagnosis and the quality of instruction. Additional directions include integrating the EKC-based profiling framework with cognitive diagnostic Computerized Adaptive Testing to reduce test length while maintaining interpretability, modeling hierarchical relations among knowledge concepts to relax independence assumptions, and validating the approach across subjects, grade levels, and assessment programs with different item formats. Future work should also examine how teachers and other assessment stakeholders interpret and use RLDM-EKC reports in practice, including agreement, usability, and decision quality under realistic classroom constraints.

Beyond statistical uncertainty, we acknowledge practical limitations and deployment obstacles. First, the model relies on conditional independence of item responses given the latent profile, and potential local dependence may affect inference in assessments with shared stimuli or testlets. Second, the profile space grows exponentially with the number of knowledge concepts, and exact enumeration may become infeasible in large-scale assessments without approximate inference or structured constraints. Third, the present validation relies on the TIMSS 2007 Grade 4 mathematics dataset; transferring the framework to other subjects, grade levels, or assessment programs may require re-aligning item–concept mappings and revisiting domain-specific assumptions. Fourth, the EKC construction and cognitive tagging process depends on the availability and quality of item metadata and expert annotations; incomplete curricular materials, inconsistent item documentation, or limited expert time can affect the fidelity of the inferred cognitive profiles. Finally, real-world adoption must comply with privacy, governance, and institutional review requirements, and the resulting feedback should be integrated into existing instructional routines in a way that is interpretable, actionable, and accountable for teachers and stakeholders.

## Figures and Tables

**Table 1:** Main symbols and their corresponding descriptions.

Symbol	Descriptions
$A_{ekc}$	Representation of the Exercise–Knowledge–Cognition tensor
*E*	Exercise space
*K*	Knowledge space
*C*	Cognitive space
$\pi_{ue}$	Ideal response of learner *u* to exercise *e*
$R_{ue}$	Actual response of learner *u* to exercise *e*
$\beta_{uk}$	Cognitive pattern of learner *u* on knowledge concept *k*
$s_{e}$	Slip parameter of exercise *e*
$g_{e}$	Guessing parameter of exercise *e*
$P_{e}\left(\beta_{n}\right)$	Correct response probability of exercise *e* under cognitive pattern $\beta_{n}$
$L\left(R|\beta \right)$	Conditional likelihood of response data *R* given cognitive pattern β
$L\left(R\right)$	Marginal likelihood of response data *R*
$P\left(\beta_{n}|R_{u}\right)$	Posterior probability that learner *u* has cognitive pattern $\beta_{n}$
$Z_{n}$	Expected number of learners with cognitive pattern $\beta_{n}$
$F_{en}$	Expected number of correct responders to exercise *e* under $\beta_{n}$
$\gamma^{final}$	Final estimated exercise parameters obtained by the EM algorithm
$\beta_{set}$	Set of learners’ cognitive patterns

**Table 2:** TIMSS 2007 sample exercise information.

Exercise ID	Subject	Grade	Domain	Cognitive Domain	Maximum Points	Key
M031286	M	4	Number	Knowing	1	See scoring guide
S032115	S	8	Data Display	Reasoning	2	A

**Table 3:** RLDM-EKC parameter estimation results and learners’ knowledge cognitive level mining results.

Exercise Number	Slip		Guess	
	True	Estimated	True	Estimated
1	0.355	0.341	0.129	0.114
2	0.249	0.122	0.269	0.231
3	0.130	0.122	0.200	0.195
4	0.195	0.247	0.108	0.118
5	0.276	0.310	0.117	0.132
6	0.119	0.096	0.160	0.176
7	0.305	0.256	0.110	0.151
8	0.149	0.131	0.257	0.231
9	0.265	0.317	0.185	0.196
10	0.265	0.304	0.271	0.227
11	0.205	0.145	0.349	0.346
12	0.137	0.151	0.356	0.363
13	0.151	0.173	0.106	0.108
14	0.232	0.249	0.264	0.284
15	0.167	0.164	0.133	0.094
16	0.143	0.181	0.233	0.274
17	0.217	0.205	0.115	0.115
18	0.192	0.200	0.202	0.178
19	0.195	0.128	0.146	0.115
20	0.211	0.222	0.186	0.195
Mean Absolute Error			0.033 (s)	0.020 (g)
Mean Square Error			0.002 (s)	0.001 (g)
Pattern Match Ratio of Learners			0.817	
Average Attribute Match Ratio of Learners			0.916	

**Table 4:** Quantized EKC tensor for TIMSS 2007 Grade 4 mathematics (excerpt).

	Number								Geometric Shapes & Measures				Data Display		
	1	2	3	4	5	6	7	8	1	2	3	4	1	2	3
M041052	2	4	0	0	0	0	0	0	0	0	0	0	0	0	0
M041056	0	0	0	0	2	0	0	0	0	0	0	0	0	0	0
M041069	0	1	0	2	2	0	0	0	0	0	0	0	0	0	0
M041258A	2	4	0	0	0	0	0	0	0	3	0	0	0	0	0
M041258B	2	4	0	0	0	0	0	0	1	3	0	0	0	0	0
…	…								…				…		
M031242A	0	1	2	0	0	0	0	3	0	0	0	0	0	0	0
M031242B	0	1	2	0	0	0	0	0	0	0	0	0	0	2	0
M031242C	0	1	2	0	0	0	0	1	0	0	0	0	0	2	0
…	…								…				…		
M031172	1	4	0	0	0	0	0	0	0	0	0	0	2	0	4

**Table 5:** Descriptive statistics of the empirical dataset.

Dastaset	Number of Learners	Number of Exercises	Number of KC
1. United States	1687	19	8
2. Minnesota	393	8	4
3. Massachusetts	381	6	3

Note: Data from the United States exclude those from Minnesota and Massachusetts.

**Table 6:** Model fit results on the TIMSS dataset.

Model	Dataset 1		Dataset 2		Dataset 3	
	AIC	BIC	AIC	BIC	AIC	BIC
RLDM-EKC	27,294	28,364	3149	3384	2394	2586
DINA	32,029	33,620	3216	3439	2587	2662
PA-DINA	31,378	31,823	3190	3309	2518	2668
pG-DINA	27,253	28,282	3170	3361	2339	2513

**Table 7:** Inferred cognitive profiles of learner No.172 in Dataset 1 by different models.

Dataset	KC	RLDM-EKC	DINA	PA-DINA	pG-DINA
1	1	Not Mastered (0)	0	0	0
	2	Applying (3)	1	3	4
	3	Evaluating (5)	1	5	5
	4	Not Mastered (0)	0	0	0
	5	Remembering (1)	0	0	0
	6	Not Mastered (0)	0	0	0
	7	Creating (6)	1	6	6
	8	Applying (3)	1	3	1
2	1	Not Mastered (0)	0	1	0
	2	Understanding (2)	0	2	2
	3	Applying (3)	1	2	2
	4	Analyzing (4)	1	3	4
3	1	Understanding (2)	1	2	1
	2	Remembering (1)	0	1	1
	3	Not Mastered (0)	0	0	0

**Table 8:** Identifiability gate and Level 2/3 adjacent-level separability for representative knowledge concepts.

Dataset	KC	Gate	$\left(n_{q2},n_{q3}\right)$	$n_{2/3}$	Indet.	Med. |log-odds|	P(|Δ|<0.10)
1	KC2	Strong	(2, 3)	388	0.090	2.251	0.003
	KC3	Weak	(6, 1)	228	1.000	0.007	0.965
	KC4	Weak	(1, 2)	888	0.034	4.682	0.016
2	KC2	Strong	(3, 3)	113	0.000	8.632	0.000
3	KC1	Weak	(3, 1)	183	1.000	0.005	1.000

**Table 9:** Distribution of item characteristics across TIMSS cognitive domains by dataset.

Dataset	TIMSS_Domain	n_Items	$c_{\max}$_Median_IQR	p_Correct_Mean
1	Knowing	5	2.0 ([2.0, 2.0])	0.279
	Applying	11	3.0 ([3.0, 4.0])	0.397
	Reasoning	3	5.0 ([5.0, 5.5])	0.259
2	Knowing	3	2.0 ([2.0, 2.0])	0.411
	Applying	5	3.0 ([3.0, 4.0])	0.419
	Reasoning	0	-	-
3	Knowing	4	2.0 ([2.0, 2.0])	0.427
	Applying	1	4.0 ([4.0, 4.0])	0.564
	Reasoning	1	5.0 ([5.0, 5.0])	0.071

**Table 10:** Results of construct validity analysis.

Dataset	Kruskal_*H*	Kruskal_*p*	$\epsilon^{2}$	Spearman_ρ	Spearman_*p*
1	15.017	$5.48\times 10^{-4}$	0.814	0.913	$4.79\times 10^{-8}$
2	5.600	0.0180	0.767	0.894	0.0027
3	5.000	0.0821	1.000	1.000	$1.85\times 10^{-32}$

## Data Availability

Data will be made available on reasonable request.

## Abbreviations

The following abbreviations are used in this manuscript:

EKC	Exercise–Knowledge–Cognition
RLDM	Refined Learning Diagnosis Model
CDM	Cognitive Diagnostic Models
MAP	Maximum A Posteriori
MCMC	Markov Chain Monte Carlo
EM	Expectation Maximization
DINA	Deterministic Inputs, Noisy “And” gate
pG-DINA	Polytomous Generalized Deterministic Inputs, Noisy, “And” gate
PA-DINA	DINA model for polytomous attributes
MAE	Mean Absolute Error
MSE	Mean Square Error
PMR	Pattern Match Ratio
AAMR	Average Attribute Match Ratio
AIC	Akaike Information Criterion
BIC	Bayesian Information Criterion
